# Hashimoto’s Thyroiditis: A Protective Factor against Recurrence in BRAF-Wild Type Differentiated Thyroid Carcinoma †

**DOI:** 10.3390/cancers15082371

**Published:** 2023-04-19

**Authors:** Peter P. Issa, Mahmoud Omar, Yusef Buti, Mohamed Aboueisha, Ruhul Munshi, Mohammad Hussein, Muhib Haidari, Graham Blair, Chad P. Issa, Mohamed Shama, Eman Toraih, Emad Kandil

**Affiliations:** 1 Department of Surgery, School of Medicine, Tulane University, New Orleans, LA 70112, USA; 2 School of Medicine, Louisiana State University Health Sciences Center, New Orleans, LA 70112, USA; 3 School of Medicine, Tulane University, New Orleans, LA 70112, USA; 4 Genetics Unit, Department of Histology and Cell Biology, Faculty of Medicine, Suez Canal University, Ismailia 41522, Egypt

**Keywords:** thyroid cancer, Hashimoto’s thyroiditis, recurrence, risk factor, protective factor

## Abstract

**Simple Summary:**

Hashimoto’s thyroiditis and BRAF-mutation are protective and risk factors for thyroid cancer aggressiveness, respectively. We assessed the influence of Hashimoto’s thyroiditis and its influence on recurrence in patients with BRAF-wild type and BRAF-mutant differentiated thyroid carcinoma. Hashimoto’s thyroiditis was determined to be an independent protective factor against recurrence only in patients with BRAF-wild type carcinomas.

**Abstract:**

A recent work analyzing the concomitant factors BRAF mutation (risk factor) and Hashimoto’s thyroiditis (HT) (protective factor) found that the presence of HT reduced lymph node metastasis in BRAF-mutated papillary thyroid carcinoma. Whether this notion is upheld with respect to disease recurrence and differentiated thyroid carcinoma (DTC), however, is unknown. We aimed to investigate the effect of underlying HT in DTC patients and its influence on recurrence with a specific emphasis in BRAF-mutated tumors. A total of 469 patients were included. Patients were stratified according to BRAF and HT status. Multivariate regression analysis was conducted to determine protective and risk factors of disease recurrence in patients with DTC. HT was associated with less-aggressive carcinomas including more frequent microcarcinomas (HT: 45.0% vs. no-HT: 34.0%, *p* = 0.02), less lymph node involvement (HT: 16.4% vs. no-HT: 26.1%, *p* = 0.02), and less disease recurrence (HT: 2.9% vs. no-HT: 11.9%, *p* = 0.002). BRAF mutation was also significantly associated with higher rates of lymph node involvement (BRAF-mutant: 41.9% vs. BRAF-wild type: 14.6%, *p* < 0.001) and almost two times the rate of recurrence (BRAF-mutant: 14.9% vs. BRAF-wild type: 6.5%, *p* = 0.004). Underlying HT was the only protective factor determined, reducing the odds of developing recurrence by 70% (HR: 0.30, 95%CI: 0.11–0.88). In the BRAF-wild type cohort, regression analysis continued to determine HT as a protective factor (*p* = 0.03). However, in the BRAF-mutant cohort, HT was no longer an independent protective factor (*p* = 0.20) against recurrence. Sub-group regression analysis, including PTC patients, similarly found HT as a protective factor only in BRAF-wild type patients (*p* = 0.039) and not BRAF-mutant (*p* = 0.627). The presence of underlying HT is associated with less aggressive tumors and is an independent protective factor against DTC recurrence, reducing the risk by 70%. HT remains a protective factor in BRAF-wild type carcinoma, but not in patients with BRAF-mutant carcinoma. HT may potentially be considered as a parameter which enhances American Thyroid Association patient risk stratification.

## 1. Introduction

Thyroid cancer is the most common endocrine malignancy and is the fastest growing cancer in the United States [1,2]. Differentiated thyroid carcinomas (DTC) are those which develop from thyroid follicular cells, including both papillary thyroid carcinomas (PTCs) and follicular thyroid carcinomas (FTCs). DTCs account for 98% of all thyroid cancers and have good prognosis [3]. Though DTC patients often have good prognosis, the rate of recurrence has been suggested to be as high as 30% in PTC patients, which comprises the majority (~80%) of DTC [4,5], warranting a demand for predictive factors for DTC aggressiveness and recurrence. One well-studied risk factor of PTC aggressiveness is BRAF V600E mutation, which is associated with higher TNM staging, decreased patient 10-year survival, as well as recurrent and persistent disease [6,7,8,9].

Hashimoto’s thyroiditis (HT) is the most common thyroid-related autoimmune disorder and is characterized by pathologic lymphocytic infiltration and resultant hypothyroidism [10]. Analogous to a two-sided sword, HT increases the risk of PTC overall while simultaneously minimizing tumor progression [6,9,11]. Though a paucity of data has investigated the notion in DTCs, patients with PTCs and underlying HT have excellent prognosis, minimizing lymph node metastasis, tumor size, and recurrence rate [12,13,14]. Therefore, underlying HT may serve as a protective factor in DTCs overall.

Our team recently analyzed the concomitant factors BRAF mutation (risk factor) and HT (protective factor) and found that the presence of HT reduced lymph node metastasis in BRAF-mutated PTCs [13]. Whether this notion is upheld with respect to disease recurrence, however, is unknown. We thought to investigate the effect of underlying HT in DTC patients and its influence on recurrence with a specific emphasis on BRAF-mutated tumors.

## 2. Methods

### 2.1. Study Design and Recruited Cohort

This retrospective study was conducted following approval by the Tulane University institutional review board. Patients undergoing thyroid surgery for the treatment of DTC between the years 2008 and 2021 were included. DTCs included PTCs and FTCs. All patients underwent thyroidectomy, including hemithyroidectomy, total thyroidectomy, total thyroidectomy with central lymph node dissection, or total thyroidectomy with both central and lateral lymph node dissection. Relevant parameters were collected, such as patient demographics, operative details, tumor cytopathological data, TNM staging, lymph node metastasis, extrathyroidal invasion, capsular invasion, and disease recurrence. All patients in this study displayed loco-regional recurrence to the central or lateral neck determined as biopsy-proven new structural disease.

### 2.2. Determination of BRAF Mutation and Hashimoto’s Thyroiditis Status

All patients included in the study were evaluated for underlying HT and BRAF mutation status. HT diagnosis was made in one of the two following scenarios. First, the patient with overt or subclinical hypothyroidism along with a moderate-to-prominent heterogeneous as well as an elevated anti-thyroid peroxidase (TPOAb; >50 U/mL) and/or elevated anti-thyroglobulin (TgAb; >40 U/mL). Second, a histopathological analysis which determined diffuse lymphocytic infiltration with lymphoid follicle formation along with reactive germinal centers, regardless of whether the patient was hypothyroid or not.

BRAF mutation analysis was conducted preoperatively or postoperatively. Preoperative samples were attained by core needle biopsy or fine-needle aspiration and evaluated by Afirma Thyroid FNA Analysis (including both GEC and GSC; Veracyte Inc., San Francisco, CA, USA) or Interpace Diagnostics ThyGenX/ThyGeNEXT/ThyraMIR (Interpace Biosciences, Parsippany, NJ, USA). Postoperative surgical specimens were analyzed for BRAF mutation by real-time polymerase chain reaction (PCR) at the University of Pittsburgh Medical Center in accordance with standard protocol. Frozen specimens which were formalin-fixed and paraffin-embedded were subject to DNA extraction using Qiagen EZ1 tissue kit (Qiagen, Hilden, Germany) following manufacture guidelines. Samples are subsequently subject to a BRAF mutation kit assessment (EntroGen, Woodland Hills, CA, USA) with a sensitivity of 1–5% in a background of wild type genomic DNA.

### 2.3. Statistical Analysis

Statistical analysis was performed using SPSS version 27.0 and SAS 9.4. Continuous variables are reported as the median and its corresponding interquartile range (IQR). Categorical variables are reported as the count and its corresponding percentage. Two-sided Chi-square, Student’s *t*, and Mann–Whitney U tests were used. A *p*-value of 0.05 was set for the determination of significance. Descriptive statistics summarizing baseline characteristics of DTC patients undergoing thyroid surgery were performed, including demographic data, patient comorbidities, pathological parameters, and disease recurrence. Stratification of patients by BRAF mutation status allowed univariate determination of the potentially harmful effect of oncogene genetic mutation. A second analysis sub-grouped by patient HT status allowed for univariate determination of the potentially protective effect of the underlying autoimmune disease. Subsequently, an additional analysis of the concomitant factors, BRAF mutation and HT, ensued to determine the extent of aggressiveness/protectiveness of each factor. Finally, regression analyses were conducted to determine protective and risk factors of disease recurrence in DTCs.

## 3. Results

### 3.1. The Study Population

The study population included 469 patients with DTC who underwent thyroid surgery (Table 1). The study cohort had a mean follow-up of 46.08 ± 58.76 months. The mean age of all patients was 50.9 ± 15.2 years and was similar between those with HT (49.9 ± 14.1 years) and without HT (51.4 ± 15.6 years) (*p* = 0.33). In addition, 29.9% (N = 140) of the population had HT and 71.1% (N = 329) did not. The population was comprised predominately of females at 76.3% (N = 358) and White patients at 65.7% of the study population. Patients with HT were significantly more likely to be female (*p* = 0.04) and White (*p* = 0.01), which is consistent with previous literature [15]. A total of 128 patients had BRAF mutation (27.3%), while 321 were BRAF wild type (68.4%).

Patients with and without HT had overall-similar risk stratification (*p* = 0.28), with the majority of patients (58.6%) characterized as low-risk. The extent of surgery patients underwent differed between patients with and without underlying HT (*p* = 0.01). Hemithyroidectomy was performed for 21.9% of patients without HT, but only in 12.9% of patients with HT. Conversely, total thyroidectomy with both central and lateral lymph node dissection was more common in patients without HT (HT: 8.6% vs. no-HT: 14.9%). Approximately one-third of patients underwent radioactive iodine (RAI) ablation therapy (HT: 30.0% vs. no-HT: 38.0%, *p* = 0.09).

Concerning pathological parameters, HT was associated with significantly less-aggressive carcinomas. The study population comprised 91% of PTC, 5.3% of FTC, and 3.6% of patients with features of both PTC and FTC. Carcinoma histopathology was not associated with HT (*p* = 0.83). The majority of patients had T1 staged carcinomas, including 69.1% of patients. Only 1.9% of patients had T4 staged tumors, with the remaining 29.0% comprised of T2 and T3 carcinomas. Patients with HT were more likely (HT: 45.0% vs. no-HT: 34.0%) to have microcarcinomas (*p* = 0.02). The presence of HT was associated with smaller tumor sizes, specifically fewer T3 (HT: 10.7% vs. no-HT:16.4%) and T4 (HT: 0.0% vs. no-HT: 2.7%) staged tumors (*p* = 0.03). HT was also significantly associated with less lymph node involvement, specifically 16.4% as compared to 26.1% in patients without HT (*p* = 0.02). Patients with HT were also significantly more likely to have less distant metastasis, including less than 1% of HT patients (0.7%) and 4.6% in non-HT patients (*p* = 0.03). Patients with HT were less likely to present with extranodal extension (HT: 4.3% vs. no-HT: 10.0%, *p* = 0.03). Importantly, patients with underlying HT had a recurrence rate of only 2.9%, which was significantly less than those without HT (11.9%, *p* = 0.002).

### 3.2. Stratification by BRAF Mutation

Patients with BRAF mutation had significantly higher risk thyroid carcinomas (Table S1). Patients with BRAF mutation were less likely to have tumors in the T1 staged tumors (BRAF-mutant: 62.8% vs. BRAF-wild type: 72.0%) and more T3 staged (BRAF-mutant: 20.9% vs. BRAF-wild type: 11.8%) and T4 staged (BRAF-mutant: 2.7% vs. BRAF-wild type: 1.6%) tumors (*p* = 0.052). BRAF mutation was also significantly associated with higher rates of lymph node involvement (BRAF-mutant: 41.9% vs. BRAF-wild type: 14.6%, *p* < 0.001), but not distant metastasis (*p* = 0.60). Importantly, patients with BRAF-mutant tumors had a recurrence rate of 14.9%, which was more than two times as common as the 6.5% observed in the BRAF-wild type cohort (*p* = 0.004).

### 3.3. Recurrence

To determine parameters associated with thyroid cancer recurrence, we stratified the population into those who demonstrated recurrence and those who did not (Table 2). A total of 43 patients had disease recurrence, comprising 9.2% of the study population. Patients with recurrence were more likely to have BRAF-mutant tumors (*p* = 0.004). Specifically, 51.2% of patients exhibiting recurrence had BRAF-mutant tumors. Importantly, patients with underlying HT had significantly less rates of recurrence (*p* = 0.002), including a total of only four patients. A single patient with underlying HT without BRAF-mutation displayed recurrence (2.3% of the population with recurrence) while three patients with recurrence had HT and BRAF-mutant tumors (7.0% of the population with recurrence). Patients with BRAF-wild type tumor and without underlying HT had similar incidences of recurrence and non-recurrence (*p* = 0.84). Similarly, patients with BRAF-mutant carcinomas with underlying HT had similar incidences of recurrence and non-recurrence (*p* = 0.57). Patients with HT and BRAF-wild type tumors were more likely to display non-recurrence (22.3% vs. 2.3%, *p* = 0.002). Patients without HT and BRAF-mutant carcinomas were more likely to exhibit disease recurrence (44.2% vs. 20.0%, *p* < 0.001).

In patients with BRAF-wild type tumors, HT was associated with decreased risk of recurrence (HT: 2.3% vs. no-HT: 46.5%, *p* = 0.009). In patients with BRAF-mutant DTC, HT tended to be associated with decreased risk of recurrence (HT: 7.0% vs. no-HT: 44.2%, *p* = 0.07).

### 3.4. Regression Analysis—Whole Cohort

**Figure 1:**
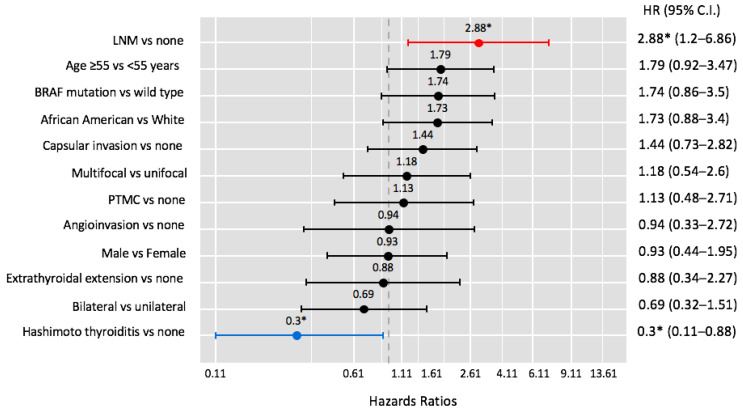
Multivariate logistic regression analysis determining independent predictors of recurrence; * indicates *p* < 0.05.

Two parameters were determined to be independent factors associated with disease recurrence. Independent predictors of disease recurrence are shown in Figure 1. Lymph node metastasis (HR: 2.88, 95%CI: 1.20–6.86) was a risk factor associated with recurrence. BRAF-mutation tended to increase the risk of recurrence by 74%, though this was statistically insignificant (HR: 1.74, 95%CI: 0.86–3.50). Underlying HT was the only protective factor determined, reducing the odds of developing recurrence by 70% (HR: 0.30, 95%CI: 0.11–0.88). A sub-group regression analysis was conducted to determine whether HT remained protective in BRAF-mutant and BRAF-wild type patients (Table 3). In the BRAF-wild type cohort, regression analysis continued to determine HT as a protective factor (*p* = 0.03). However, in the BRAF-mutant cohort, HT was no longer an independent protective factor (*p* = 0.20) against recurrence.

### 3.5. Regression Analysis—PTC Patients

Since BRAF mutation is a significant driver mutation and associated with PTC, a sub-group analysis including only patients with PTC was conducted (Table 4). In this sub-population, HT and BRAF-mutation each only tended to decrease and increase the risk of recurrence, respectively. HT tended to reduce the risk of recurrence by 65% (HR: 0.35, 95%CI: 0.12–1.032), while BRAF mutation tended to increase the risk of recurrence (HR: 1.94, 95%CI: 0.94–3.99). Given the limited patient population experienced recurrence, it is worth noting that this sub-group analysis may have been underpowered to elicit a statistical significance.

A second sub-group analysis was conducted to elucidate whether HT or other parameters were independent predictors of recurrence with respect to BRAF mutation status (Table 5). Two parameters, one risk factor and one protective factor, were elicited on multivariate regression analysis of BRAF-wild type PTC. Lymph node metastasis increased the risk of recurrence by greater than 4-fold (HR: 4.12, 95%CI: 1.15–14.78), while HT reduced the risk of recurrence by almost 90% (HR: 1.94, 95%CI: 0.01–0.89). In BRAF-mutant PTC, however, neither lymph node metastasis (HR: 1.34, 95%CI: 0.35–5.09) nor HT (HR: 0.69, 95%CI: 0.16–3.03) remained independent predictors of recurrence. These findings were consistent with regression analyses of the overall study population.

## 4. Discussion

Thyroid cancer is the fastest growing cancer in the United States, largely attributed to increased surveillance and detection rates. The most recent 2015 revision of the ATA guidelines cautions against overdiagnosis and overtreatment [16]. BRAF is a common mutation associated with increased tumor aggressiveness, including higher TNM staging and increased recurrence rates [6,7,8,9]. HT is the most common reason for hypothyroidism and is an autoimmune disease associated with chronic inflammation. Though works have suggested BRAF mutation as a risk factor for tumor aggressiveness and HT as a protective factor against tumor aggressiveness, few works have investigated the two simultaneously [11,17]. One potential explanation for the protective effect of HT, however, is the increased medical attention these patients already receive, and consequently, the detection of carcinomas earlier. To our best knowledge, this is the first work to investigate the risk-reducing effect of HT in BRAF-mutant DTC. Our work suggests HT as a protective factor against DTC risk of recurrence only in patients with BRAF-wild type carcinomas.

The coexistence of HT and thyroid carcinoma is not uncommon, with a range of 14.2–37.7% [18,19,20]. Interestingly, several works have suggested that nearly half of patients diagnosed with PTC have underlying HT [17,19,21]. For example, a 2017 meta-analysis of 27 studies (N = 76,821) reported two times (OR = 2.12, 95%CI: 1.78–2.52) the risk of PTC in HT patients as opposed to non-HT patients [15]. The etiology and pathophysiological mechanism underlying this phenomenon are still not well understood. One hypothesis suggests HT is a consequence of the initially present carcinoma, with malignancy inducing a sustained immune response stimulating lymphocytic infiltration of the thyroid gland and consequent HT [22]. Conversely, carcinoma may develop in response to the initially present HT, with chronic inflammation inducing a favorable environment for malignant transformation and subsequent dysregulation of the follicular cells [23]. In addition, destructive hypothyroidism in the setting of TSH stimulation (hypothyroidism) may further stimulate follicular proliferation and hyperplasia, which promotes carcinogenesis [24]. Irrespective of etiology, which may be a combination of both hypotheses, multiple works have suggested that HT is associated with less aggressive tumor stage and lower rates of recurrence [25,26,27]. For example, Dvorkin et al. reported smaller primary tumors (17.9 vs. 21.2 mm, *p* = 0.01), less lymph node metastasis (23% vs. 34%, *p* = 0.02), and higher rates of no evidence of disease at final follow-up (87.7% vs. 76.2%, *p* = 0.02) [26]. Our work similarly demonstrated decreased lymph node metastasis, extranodal extension, and distant metastasis in patients with underlying HT, suggesting underlying HT as a protective factor against DTC risk.

DTC includes both PTC and FTC and has a relatively favorable prognosis in comparison to other forms of thyroid malignancy with a low disease-specific mortality rate [28]. Still, multiple works have reported worrisome rates of recurrence in DTC patients ranging from 10% to 43.5% [29,30,31]. One single-institution experience of 2444 PTC reported a recurrence rate of 14% over 25 years [32]. Other studies cited recurrence rates up to 30% [32,33]. Witte et al. reported the rate of recurrence in FTC in their cohort to be 43.5%, with the majority of these recurrences occurring within the first 3 years following surgery [31]. We found an overall rate of recurrence of 9.2%, the majority of these (90.7%) being those without underlying HT. It is worth noting that a considerable portion of the DTC in our study was T1 (69.1%), potentially suggesting the overdiagnosis and treatment of low-risk carcinoma, which are less likely to exhibit recurrence. On multivariate regression analysis, HT was the only protective factor, reducing the risk of recurrence by 70%. Importantly, HT continued to be a protective factor only in the BRAF-wild type cohort, but could not elicit a protective effect in the BRAF-mutant group. A similar situation was upheld in the sub-group analyses including only patients with PTC, where HT was a protective factor only in the BRAF-mutant PTC cohort. Several works have investigated the overall role of HT and reported similar findings, including the works of Loh et al. and Huang et al. who found 24% versus 6% and 53.2% versus 0% rates of recurrence in non-HT and HT patients, respectively [34,35]. Importantly, our work allows an understanding of the extent of the protective ability of HT as our cohort analyzed the risk of recurrence in the whole patient population as well as those with and without underlying HT. In BRAF-wild type DTC, HT was a protective factor. In BRAF-mutant patients, HT only tended to decrease the risk of recurrence (44.2% of recurrences were BRAF-mutant without HT, 7.0% of recurrences were BRAF-mutant with HT). Considering the ATA’s patient risk stratification, which is based on recurrence, HT could potentially be considered as a parameter which enhances risk stratification.

BRAF mutation is a commonly mutated oncogene in thyroid cancer associated with advanced tumor progression and decreased 10-year survival [36,37,38]. A plethora of literature suggests BRAF mutation in DTC increases the risk of multifocality, extrathyroidal extension, and lymph node metastasis [39,40,41]. Importantly, patients with BRAF-mutant PTC are almost two times as likely (RR = 1.90, 95%CI: 1.43–2.53) to experience disease recurrence [38]. Still, only a few works have investigated the co-existence of BRAF mutation and HT. It has been suggested that BRAF mutation is less common among patients with HT [42,43]. For example, Kim et al. in their study, including 101 PTC, reported BRAF mutation to be more frequent (95.3% vs. 72.9%, *p* = 0.003) in the cohort without Hashimoto’s thyroiditis [43]. Another work from Korea reported similar findings in their study of 3332 PTC (76.9% vs. 86.6%, *p* < 0.001) [42]. While the incidence of BRAF-mutated PTC was high in this study, multiple works from South Korea have reported similar ranges of BRAF mutation from 73.4% to 86% [44,45,46]. Importantly, one study including 146 PTCs, of which 116 were BRAF-mutant, suggested HT to serve as a protective factor even in the setting of BRAF mutation [11]. The authors reported decreased rates of extracapsular extension (57.6% vs. 29.6%, *p* = 0.001) and smaller primary tumor sizes (T1 staging 77.8% vs. 60.8%, *p* = 0.028). Another work including 3332 PTC (83.7% BRAF-mutated, N = 2789), found HT to serve as an independent predictor of decreased risk of both extrathyroidal extension and central lymph node metastasis in patients with and without BRAF-mutant tumors [42]. The authors did not investigate the risk of recurrence in their cohort [42]. In our study, a total of four patients with HT experienced disease recurrence (N = 4/43, 9.3% of all patients with recurrence), with only a single patient (N = 1/43, 2.3% of all patients with recurrence) having HT and a BRAF-wild type carcinoma. In our study, HT was determined to be a protective factor when considering the whole study population. Importantly, however, HT was only a protective factor in patients with BRAF-wild type carcinomas, but not the BRAF-mutant subpopulation. While future studies with larger cohorts are warranted to corroborate these findings, our work suggests HT to be an independent protective factor against disease recurrence in patients with BRAF-wild type DTC.

DTC recurrence is associated with advanced disease. For example, tumor-specific factors predicting recurrence include tumor maximal diameter, higher T stage, presence of extrathyroidal extension, aggressive histological tumor, and positive lymph node involvement at the time of diagnosis [47,48,49]. These tumor-specific factors are often found at advanced/higher stages in patients with BRAF-mutant carcinomas and lower stages in patients with underlying HT. It is worth noting, however, that one potential explanation for this could be increased surveillance of patients with HT allowing earlier detection, and consequently, less-aggressive malignancies [50].

Current American Thyroid Association (ATA) guidelines specify BRAF mutation as a parameter associated with a higher risk of recurrence [16]. However, the guidelines do not specify or highlight the potential role of HT as a protective factor. While future studies are warranted to validate our findings, patients with underlying HT may be candidates to potentially be treated appropriately with less aggressive management options. Less aggressive management is associated with less complication [51,52]. For patients with small PTC or both small PTC and underlying HT, minimally-invasive treatment options, such as radiofrequency ablation (RFA) may be an appropriate management option. Several works, including a prospective multi-institutional work of our own, have demonstrated RFA to be a safe and effective modality for the management of benign thyroid nodules and PTC [53,54,55]. More recently, however, RFA has been reported to be both safe and efficacious for PTC patients with HT [56,57]. For patients with small PTC preferring to minimize treatment aggressiveness, active surveillance (non-surgical management) may be an appropriate treatment, as well [58]. Given the protective nature of HT, medical management may be an especially suitable option for this patient population with small PTC without complaint of symptomatic hypothyroidism. Since HT can counteract the effect of BRAF mutation and neutralize the risk of lymph node metastasis in PTC patients [17], this specific subset of patients may maintain treatment as if harboring a wild type PTC.

Our study has strengths and limitations. One limitation of the study includes the mean follow-up of just under four years. A previous work including 1020 PTC patients reported that recurrence all incidencies of recurrence occurred within the first 8 years of follow-up, 76.9% within 5 years, and 46.2% within 3 years [59]. Therefore, while our study captures the majority of patients who would demonstrate recurrence, future studies are warranted with longer follow-up to determine a long-term perspective. Another limitation is the retrospective nature of the study which allows inherent biases. One other limitation is the limited population of patients with BRAF-mutant tumors who exhibited disease recurrence, which may have allowed for an underpowered analysis. Future investigation with larger sample sizes are necessary to corroborate our findings. One strength of the study was the large overall and racially-diverse patient population, which allows for the generalizability of the data.

## 5. Conclusions

The presence of underlying HT is associated with less aggressive tumors and is an independent protective factor against DTC recurrence, reducing the risk by 70%. HT remains a protective factor in BRAF-wild type DTC, but not in patients with BRAF-mutant DTC. HT may potentially be considered as a parameter which enhances ATA patient risk stratification.

## Figures and Tables

**Table 1:** Baseline characteristics of differentiated thyroid cancer patients who underwent thyroid surgery with subgroup analysis based on underlying Hashimoto’s thyroiditis. Data are presented as number and percentage, mean and standard deviation, or median and interquartile range. Percentage is reported per the designated group. Two-sided Chi-Square, Student’s *t*, and Mann–Whitney U tests were used. The study cohort had a mean follow-up of 46.08 ± 58.76 months. ATA: American Thyroid Association, RAI: Radioactive iodine, PTMC: Papillary thyroid micro-carcinoma, PTC: Papillary thyroid carcinoma, FTC: Follicular thyroid carcinoma, TT: Total thyroidectomy, CLND: Central lymph node dissection, LLND: Lateral lymph node dissection, SD: Standard deviation, IQR: Interquartile range.

Characteristics	Levels	Total	No Hashimoto’s Thyroiditis	Hashimoto’s Thyroiditis	*p*-Value
**Number**		469	329 (70.1%)	140 (29.9%)	
**Demographic data**					
Age	Mean (SD)	50.9 (15.2)	51.4 (15.6)	49.9 (14.1)	0.33
Gender	Female	358 (76.3%)	239 (72.6%)	119 (85.0%)	0.04
	Male	111 (23.7%)	90 (27.4%)	21 (15.0%)	
Race	White	308 (65.7%)	205 (62.3%)	103 (73.6%)	0.01
	African American	161 (34.3%)	124 (37.7%)	37 (26.4%)	
**Management**					
ATA Risk Group	Low	275 (58.6%)	188 (57.1%)	87 (62.1%)	0.28
	Intermediate	137 (29.2%)	96 (29.2%)	41 (29.3%)	
	High	57 (12.2%)	45 (13.7%)	12 (8.6%)	
Type of Surgery	Hemithyroidectomy	90 (19.2%)	72 (21.9%)	18 (12.9%)	0.01
	TT	216 (46.1%)	141 (42.9%)	75 (53.6%)	
	TT with CLND	102 (21.7%)	67 (20.4%)	35 (25.0%)	
	TT with CLND + LLND	61 (13.0%)	49 (14.9%)	12 (8.6%)	
RAI treatment	Positive	167 (35.6%)	125 (38.0%)	42 (30.0%	0.09
Follow-Up	Months (IQR)	25.22 (6.01–59.22)	26.43 (6.63–62.82)	24.15 (5.39–55.43)	0.453
**Pathological data**					
Tumor type	PTC	427 (91.0%)	299 (90.9%)	128 (91.4%))	0.83
	FTC	25 (5.3%)	17 (5.2%)	8 (5.7%)	
	PTC + FTC	17 (3.6%)	13 (4.0%)	4 (2.9%)	
PTMC	Positive	175 (37.3%)	112 (34.0%)	63 (45.0%)	0.02
T stage	T1	324 (69.1%)	225 (68.4)	99 (70.7)	0.03
	T2	67 (14.3)	41 (12.5)	26 (18.6)	
	T3	69 (14.7)	54 (16.4)	15 (10.7	
	T4	9 (1.9)	9 (2.7)	0 (0)	
N stage	N0	360 (76.8%)	243 (73.9%)	117 (83.6%)	0.02
	N1	109 (23.2%)	86 (26.1%)	23 (16.4%)	
M stage	M0	453 (96.6%)	314 (95.4%)	139 (99.3%)	0.03
	M1	16 (3.4%)	15 (4.6%)	1 (0.7%)	
Extrathyroidal extension	Positive	54 (11.5%)	43 (13.1%)	11 (7.9%)	0.10
Angioinvasion	Positive	48 (10.2%)	37 (11.2%)	11 (7.9%)	0.26
Perineural invasion	Positive	5 (1.1%)	3 (0.9%)	2 (1.4%)	0.61
Capsular invasion	Positive	126 (26.9%)	86 (26.1%)	40 (28.6%)	0.58
Extranodal extension	Positive	39 (8.3%)	33 (10.0%)	6 (4.3%)	0.03
Central lymph node metastasis	Positive	106 (22.6%)	83 (25.2%)	23 (16.4%)	0.03
Lateral lymph node metastasis	Positive	65 (13.9%)	54 (16.4%)	11 (7.9%)	0.01
**Gene Mutation**					
Mutation	BRAF	148 (31.6%)	104 (31.6%)	44 (31.4%)	0.96
**Outcomes**					
Recurrence	Negative	426 (90.8%)	290 (88.1%)	136 (97.1%)	0.002
	Positive	43 (9.2%)	39 (11.9%)	4 (2.9%)	

**Table 2:** Recurrence and non-recurrence in patients by Hashimoto’s thyroiditis and BRAF mutation. Percentage is reported per the designated group. Data are presented as number and percentage. Two-sided Chi-Square, Student’s *t*, and Mann–Whitney U tests were used.

Characteristics	Non-Recurrence	Recurrence	*p*-Value
**Number**	426	43	
**Parameters**			
Hashimoto’s thyroiditis	136 (31.9%)	4 (9.3%)	0.002
BRAF mutation	126 (29.6%)	22 (51.2%)	0.004
**Pathological groups**			
Hashimoto negative, BRAF negative	205 (48.1%)	20 (46.5%)	0.84
Hashimoto positive, BRAF negative	95 (22.3%)	1 (2.3%)	0.002
Hashimoto negative, BRAF positive	85 (20.0%)	19 (44.2%)	<0.001
Hashimoto positive, BRAF positive	41 (9.6%)	3 (7.0%)	0.57

**Table 3:** Multivariate logistic regression analysis determining independent predictors of recurrence sub-grouped by BRAF mutation status.

BRAF-Wild Type	Variable	*p*-Value	HR	Lower CI	Upper CI
	Age < 55	0.141	2.215	0.769	6.377
	Male	0.909	1.071	0.330	3.472
	AA race	0.925	0.951	0.335	2.702
	**Hashimoto**	0.030	0.097	0.012	0.799
	PTMC	0.571	0.690	0.191	2.493
	Focality	0.457	0.614	0.170	2.219
	Laterality	0.220	0.358	0.070	1.845
	**LNM**	0.000	10.778	3.247	35.775
	ETE	0.132	2.854	0.730	11.158
**BRAF-mutant**	**Variable**	***p*-value**	**HR**	**Lower CI**	**Upper CI**
	Age < 55	0.815	0.885	0.317	2.467
	Male	0.970	1.022	0.327	3.190
	AA race	0.594	1.326	0.470	3.740
	HT	0.204	0.403	0.099	1.637
	PTMC	0.699	1.311	0.332	5.179
	Focality	0.179	2.306	0.682	7.802
	Laterality	0.548	1.428	0.447	4.563
	LNM	0.084	3.041	0.862	10.726
	ETE	0.896	0.924	0.284	3 006

**Table 4:** Multivariate logistic regression analysis including only PTC patients to determine independent predictors of recurrence.

Variable	*p*-Value	HR	Lower CI	Upper CI
BRAF-wild type	0.073	1.937	0.940	3.992
ETE	0.620	0.782	0.296	2.067
LNM	0.102	2.152	0.860	5.386
Age < 55	0.117	1.717	0.874	3.375
AA race	0.066	1.926	0.958	3.869
Capsular Invasion	0.272	1.478	0.737	2.967
Locality	0.785	1.119	0.498	2.514
PTMC	0.940	0.967	0.401	2.330
Angioinvasion	0.607	0.713	0.196	2.589
Male	0.881	0.942	0.435	2.042
Laterality	0.393	0.703	0.314	1.576
**HT**	0.057	0.353	0.121	1.032

**Table 5:** Multivariate logistic regression analysis including only PTC patients to determine independent predictors of recurrence sub-grouped by BRAF mutation status.

BRAF-Wild Type	Variable	*p*-Value	HR	Lower CI	Upper CI
	ETE	0.802	1.239	0.233	6.599
	**LNM**	0.030	4.117	1.147	14.778
	Age < 55	0.056	2.925	0.973	8.795
	AA race	0.948	1.037	0.348	3.090
	Capsular Invasion	0.902	0.916	0.227	3.705
	Locality	0.471	0.574	0.127	2.591
	PTMC	0.384	0.559	0.151	2.067
	Angioinvasion	0.654	0.557	0.043	7.211
	Male	0.620	1.388	0.379	5.079
	Laterality	0.341	0.337	0.036	3.162
	**HT**	0.039	0.102	0.012	0.894
**BRAF-mutant**	**Variable**	***p*-value**	**HR**	**Lower CI**	**Upper CI**
	ETE	0.227	0.415	0.100	1.728
	LNM	0.665	1.343	0.354	5.093
	Age < 55	0.797	1.138	0.425	3.045
	AA race	0.067	2.882	0.930	8.930
	Capsular Invasion	0.153	1.954	0.779	4.898
	Locality	0.152	2.592	0.704	9.549
	PTMC	0.496	1.584	0.422	5.954
	Angioinvasion	0.837	1.210	0.198	7.381
	Male	0.928	1.055	0.334	3.329
	Laterality	0.467	0.650	0.204	2.075
	HT	0.627	0.694	0.159	3.030

## Data Availability

Data are contained within the article.

## Supplementary Materials

The following supporting information can be downloaded at: https://www.mdpi.com/article/10.3390/cancers15082371/s1, Supplemental Table S1. Baseline characteristics of differentiated thyroid cancer patients who underwent thyroid surgery with subgroup analysis based on genetic mutation status. Two-sided Chi-Square, Student’s *t*, and Mann-Whitney U tests were used.

## References

[1] Morris L.G., Sikora A.G., Tosteson T.D., Davies L. (2013). The increasing incidence of thyroid cancer: The influence of access to care. Thyroid.

[2] Rossi E.D., Pantanowitz L., Hornick J.L. (2021). A worldwide journey of thyroid cancer incidence centred on tumour histology. Lancet Diabetes Endocrinol..

[3] Kebebew E., Clark O.H. (2000). Differentiated thyroid cancer:“complete” rational approach. World J. Surg..

[4] Czarniecka A., Oczko-Wojciechowska M., Barczyński M. (2016). BRAF V600E mutation in prognostication of papillary thyroid cancer (PTC) recurrence. Gland. Surg..

[5] Omry-Orbach G. (2016). Risk stratification in differentiated thyroid cancer: An ongoing process. Rambam Maimonides Med. J..

[6] Kebebew E., Weng J., Bauer J., Ranvier G., Clark O.H., Duh Q.-Y., Shibru D., Bastian B., Griffin A. (2007). The prevalence and prognostic value of BRAF mutation in thyroid cancer. Ann. Surg..

[7] Kim T.H., Park Y.J., Lim J.A., Ahn H.Y., Lee E.K., Lee Y.J., Kim K.W., Hahn S.K., Youn Y.K., Kim K.H. (2012). The association of the BRAFV600E mutation with prognostic factors and poor clinical outcome in papillary thyroid cancer: A meta-analysis. Cancer.

[8] Elisei R., Ugolini C., Viola D., Lupi C., Biagini A., Giannini R., Romei C., Miccoli P., Pinchera A., Basolo F. (2008). BRAFV600E mutation and outcome of patients with papillary thyroid carcinoma: A 15-year median follow-up study. J. Clin. Endocrinol. Metab..

[9] Xing M. (2009). BRAF mutation in papillary thyroid microcarcinoma: The promise of better risk management. Ann. Surg. Oncol..

[10] Antonelli A., Ferrari S.M., Corrado A., Di Domenicantonio A., Fallahi P. (2015). Autoimmune thyroid disorders. Autoimmun. Rev..

[11] Marotta V., Guerra A., Zatelli M.C., Uberti E.D., Di Stasi V., Faggiano A., Colao A., Vitale M. (2013). BRAF mutation positive papillary thyroid carcinoma is less advanced when H ashimoto’s thyroiditis lymphocytic infiltration is present. Clin. Endocrinol..

[12] Kashima K., Yokoyama S., Noguchi S., Murakami N., Yamashita H., Watanabe S., Uchino S., Toda M., Sasaki A., Daa T. (1998). Chronic thyroiditis as a favorable prognostic factor in papillary thyroid carcinoma. Thyroid.

[13] Zeng R., Jin L., Chen E., Dong S., Cai Y., Huang G., Li Q., Jin C., Zhang X., Wang O. (2016). Potential relationship between Hashimoto’s thyroiditis and BRAFV600E mutation status in papillary thyroid cancer. Head Neck.

[14] Battistella E., Pomba L., Costantini A., Scapinello A., Toniato A. (2022). Hashimoto’s Thyroiditis and Papillary Cancer Thyroid Coexistence Exerts a Protective Effect: A Single Centre Experience. Indian J. Surg. Oncol..

[15] Lai X., Xia Y., Zhang B., Li J., Jiang Y. (2017). A meta-analysis of Hashimoto’s thyroiditis and papillary thyroid carcinoma risk. Oncotarget.

[16] Haugen B.R., Alexander E.K., Bible K.C., Doherty G.M., Mandel S.J., Nikiforov Y.E., Pacini F., Randolph G.W., Sawka A.M., Schlumberger M. (2016). 2015 American Thyroid Association management guidelines for adult patients with thyroid nodules and differentiated thyroid cancer: The American Thyroid Association guidelines task force on thyroid nodules and differentiated thyroid cancer. Thyroid.

[17] Issa P.P., Omar M., Buti Y., Issa C.P., Chabot B., Carnabatu C.J., Munshi R., Hussein M., Aboueisha M., Shama M. (2022). Hashimoto’s Thyroiditis Minimizes Lymph Node Metastasis in BRAF Mutant Papillary Thyroid Carcinomas. Biomedicines.

[18] Repplinger D., Bargren A., Zhang Y.-W., Adler J.T., Haymart M., Chen H. (2008). Is Hashimoto’s thyroiditis a risk factor for papillary thyroid cancer?. J. Surg. Res..

[19] Zhang Y., Dai J., Wu T., Yang N., Yin Z. (2014). The study of the coexistence of Hashimoto’s thyroiditis with papillary thyroid carcinoma. J. Cancer Res. Clin. Oncol..

[20] Mukasa K., Noh J.Y., Kunii Y., Matsumoto M., Sato S., Yasuda S., Suzuki M., Ito K., Ito K. (2011). Prevalence of malignant tumors and adenomatous lesions detected by ultrasonographic screening in patients with autoimmune thyroid diseases. Thyroid.

[21] Tamimi D.M. (2002). The association between chronic lymphocytic thyroiditis and thyroid tumors. Int. J. Surg. Pathol..

[22] Büyükaşık O., Hasdemir A.O., Yalçın E., Celep B., Şengül S., Yandakçı K., Tunç G., Küçükpınar T., Alkoy S., Çöl C. (2011). The association between thyroid malignancy and chronic lymphocytic thyroiditis: Should it alter the surgical approach?. Endokrynol. Pol..

[23] Okayasu I., Fujiwara M., Hara Y., Tanaka Y., Rose N.R. (1995). Association of chronic lymphocytic thyroiditis and thyroid papillary carcinoma. A study of surgical cases among Japanese, and white and African Americans. Cancer.

[24] Lee J.-H., Kim Y., Choi J.-W., Kim Y.-S. (2013). The association between papillary thyroid carcinoma and histologically proven Hashimoto’s thyroiditis: A meta-analysis. Eur. J. Endocrinol..

[25] Dvorkin S., Robenshtok E., Hirsch D., Strenov Y., Shimon I., Benbassat C.A. (2013). Differentiated thyroid cancer is associated with less aggressive disease and better outcome in patients with coexisting Hashimotos thyroiditis. J. Clin. Endocrinol. Metab..

[26] Moon S., Chung H.S., Yu J.M., Yoo H.J., Park J.H., Kim D.S., Park Y.J. (2018). Associations between Hashimoto thyroiditis and clinical outcomes of papillary thyroid cancer: A meta-analysis of observational studies. Endocrinol. Metab..

[27] Nixon I.J., Ganly I., Palmer F.L., Whitcher M.M., Patel S.G., Tuttle R.M., Shaha A.R., Shah J.P. (2011). Disease-related death in patients who were considered free of macroscopic disease after initial treatment of well-differentiated thyroid carcinoma. Thyroid.

[28] Chrisoulidou A., Boudina M., Tzemailas A., Doumala E., Iliadou P.K., Patakiouta F., Pazaitou-Panayiotou K. (2011). Histological subtype is the most important determinant of survival in metastatic papillary thyroid cancer. Thyroid Res..

[29] Guo K., Wang Z. (2014). Risk factors influencing the recurrence of papillary thyroid carcinoma: A systematic review and meta-analysis. Int. J. Clin. Exp. Pathol..

[30] Witte J., Goretzki P.E., Dieken J., Simon D., Röher H.D. (2002). Importance of lymph node metastases in follicular thyroid cancer. World J. Surg..

[31] Hay I.D., Thompson G.B., Grant C.S., Bergstralh E.J., Dvorak C.E., Gorman C.A., Maurer M.S., McIver B., Mullan B.P., Oberg A.L. (2002). Papillary thyroid carcinoma managed at the Mayo Clinic during six decades (1940–1999): Temporal trends in initial therapy and long-term outcome in 2444 consecutively treated patients. World J. Surg..

[32] Shen W.T., Ogawa L., Ruan D., Suh I., Kebebew E., Duh Q.-Y., Clark O.H. (2010). Central neck lymph node dissection for papillary thyroid cancer: Comparison of complication and recurrence rates in 295 initial dissections and reoperations. Arch. Surg..

[33] Huang B.-Y., Hseuh C., Chao T.-C., Lin K.-J., Lin J.-D. (2011). Well-differentiated thyroid carcinoma with concomitant Hashimoto’s thyroiditis present with less aggressive clinical stage and low recurrence. Endocr. Pathol..

[34] Loh K.-C., Greenspan F.S., Dong F., Miller T.R., Yeo P.P. (1999). Influence of lymphocytic thyroiditis on the prognostic outcome of patients with papillary thyroid carcinoma. J. Clin. Endocrinol. Metab..

[35] Xing M., Alzahrani A.S., Carson K.A., Viola D., Elisei R., Bendlova B., Yip L., Mian C., Vianello F., Tuttle R.M. (2013). Association between BRAF V600E mutation and mortality in patients with papillary thyroid cancer. JAMA.

[36] Xing M. (2007). BRAF mutation in papillary thyroid cancer: Pathogenic role, molecular bases, and clinical implications. Endocr. Rev..

[37] Attia A.S., Hussein M., Issa P.P., Elnahla A., Farhoud A., Magazine B.M., Youssef M.R., Aboueisha M., Shama M., Toraih E. (2022). Association of BRAFV600E Mutation with the Aggressive Behavior of Papillary Thyroid Microcarcinoma: A Meta-Analysis of 33 Studies. Int. J. Mol. Sci..

[38] Xing M., Alzahrani A.S., Carson K.A., Shong Y.K., Kim T.Y., Viola D., Elisei R., Bendlová B., Yip L., Mian C. (2015). Association between BRAF V600E mutation and recurrence of papillary thyroid cancer. J. Clin. Oncol..

[39] Howell G.M., Nikiforova M.N., Carty S.E., Armstrong M.J., Hodak S.P., Stang M.T., McCoy K.L., Nikiforov Y.E., Yip L. (2013). BRAF V600E mutation independently predicts central compartment lymph node metastasis in patients with papillary thyroid cancer. Ann. Surg. Oncol..

[40] Subash A., Sinha P., Singh A. (2020). BRAF mutation and age in differentiated thyroid cancer risk stratification: Two sides of the same coin. Oral Oncol..

[41] Kim S.K., Woo J.-W., Lee J.H., Park I., Choe J.-H., Kim J.-H., Kim J.S. (2016). Chronic lymphocytic thyroiditis and BRAF V600E in papillary thyroid carcinoma. Endocr. Relat. Cancer.

[42] Kim S.K., Song K.-H., Lim S.D., Lim Y.C., Yoo Y.B., Kim J.S., Hwang T.S. (2009). Clinical and pathological features and the BRAF V600E mutation in patients with papillary thyroid carcinoma with and without concurrent Hashimoto thyroiditis. Thyroid.

[43] Kim K.H., Kang D.W., Kim S.H., Seong I.O., Kang D.Y. (2004). Mutations of the BRAF gene in papillary thyroid carcinoma in a Korean population. Yonsei Med. J..

[44] Chung K., Yang S.K., Lee G.K., Kim E.Y., Kwon S., Lee S.H., Park D.J., Lee H.S., Cho B.Y., Lee E.S. (2006). Detection of BRAFV600E mutation on fine needle aspiration specimens of thyroid nodule refines cyto-pathology diagnosis, especially in BRAFV600E mutation-prevalent area. Clin. Endocrinol..

[45] Kim T.Y., Kim W.B., Rhee Y.S., Song J.Y., Kim J.M., Gong G., Lee S., Kim S.Y., Kim S.C., Hong S.J. (2006). The BRAF mutation is useful for prediction of clinical recurrence in low-risk patients with conventional papillary thyroid carcinoma. Clin. Endocrinol..

[46] Baek S.-K., Jung K.-Y., Kang S.-M., Kwon S.-Y., Woo J.-S., Cho S.-H., Chung E.-J. (2010). Clinical risk factors associated with cervical lymph node recurrence in papillary thyroid carcinoma. Thyroid.

[47] Palme C.E., Waseem Z., Raza S.N., Eski S., Walfish P., Freeman J.L. (2004). Management and outcome of recurrent well-differentiated thyroid carcinoma. Arch. Otolaryngol. Neck Surg..

[48] Beasley N.J., Lee J., Eski S., Walfish P., Witterick I., Freeman J.L. (2002). Impact of nodal metastases on prognosis in patients with well-differentiated thyroid cancer. Arch. Otolaryngol. Neck Surg..

[49] Xu J., Ding K., Mu L., Huang J., Ye F., Peng Y., Guo C., Ren C. (2022). Hashimoto’s Thyroiditis: A “Double-Edged Sword” in Thyroid Carcinoma. Front. Endocrinol..

[50] Larson S.D., Jackson L.N., Riall T.S., Uchida T., Thomas R.P., Qiu S., Evers B.M. (2007). Increased incidence of well-differentiated thyroid cancer associated with Hashimoto thyroiditis and the role of the PI3k/Akt pathway. J. Am. Coll. Surg..

[51] Baek J.H., Lee J.H., Sung J.Y., Bae J.-I., Kim K.T., Sim J., Baek S.M., Kim Y., Shin J.H., Park J.S. (2012). Complications encountered in the treatment of benign thyroid nodules with US-guided radiofrequency ablation: A multicenter study. Radiology.

[52] Hsiao V., Light T.J., Adil A.A., Tao M., Chiu A.S., Hitchcock M., Arroyo N., Fernandes-Taylor S., Francis D.O. (2022). Complication Rates of Total Thyroidectomy vs Hemithyroidectomy for Treatment of Papillary Thyroid Microcarcinoma: A Systematic Review and Meta-analysis. JAMA Otolaryngol. Neck Surg..

[53] Kandil E., Omar M., Aboueisha M., Attia A.S., Ali K.M., RF A.A., Issa P.P., Wolfe S., Omari S., Buti Y. (2022). Efficacy and Safety of Radiofrequency Ablation of Thyroid Nodules: A Multi-institutional Prospective Cohort Study. Ann. Surg..

[54] Issa P.P., Omar M., Issa C.P., Buti Y., Hussein M., Aboueisha M., Abdelhady A., Shama M., Lee G.S., Toraih E. (2022). Radiofrequency Ablation of Indeterminate Thyroid Nodules: The First North American Comparative Analysis. Int. J. Mol. Sci..

[55] Van Dijk S.P., Coerts H.I., Gunput S.T., Van Velsen E.F., Medici M., Moelker A., Peeters R.P., Verhoef C., Van Ginhoven T.M. (2022). Assessment of radiofrequency ablation for papillary microcarcinoma of the thyroid: A systematic review and meta-analysis. JAMA Otolaryngol. Neck Surg..

[56] Lai L., Liu Z., Zhang J., Ni X., Liu J., Luo T., Dong Y., Zhou J. (2022). Effect of Hashimoto’s thyroiditis on the extent of the ablation zone in early stages of ultrasound-guided radiofrequency ablation for papillary thyroid microcarcinoma: A large cohort study of 772 patients. Int. J. Hyperth..

[57] Zhang Y., Zhang M., Luo Y., Li J., Zhang Y., Tang J. (2019). Effect of chronic lymphocytic thyroiditis on the efficacy and safety of ultrasound-guided radiofrequency ablation for papillary thyroid microcarcinoma. Cancer Med..

[58] Tuttle R.M., Fagin J.A., Minkowitz G., Wong R.J., Roman B., Patel S., Untch B., Ganly I., Shaha A.R., Shah J.P. (2017). Natural history and tumor volume kinetics of papillary thyroid cancers during active surveillance. JAMA Otolaryngol. Neck Surg..

[59] Durante C., Montesano T., Torlontano M., Attard M., Monzani F., Tumino S., Costante G., Meringolo D., Bruno R., Trulli F. (2013). Papillary thyroid cancer: Time course of recurrences during postsurgery surveillance. J. Clin. Endocrinol. Metab..

