# Supplementary material for: Hashimoto’s Thyroiditis: A Protective Factor against Recurrence in BRAF-Wild Type Differentiated Thyroid Carcinoma
Source: Cancers (Basel). 2023 Apr 19;15(8):2371. doi: 10.3390/cancers15082371 (PMC10137168; doi:10.3390/cancers15082371)
Supplement: Supplementary file 1 [file cancers-15-02371-s001.zip › cancers-2299542-supplementary.pdf]

Supplemental Table S1. Baseline characteristics of differentiated thyroid cancer patients who underwent thyroid surgery with subgroup analysis based on genetic mutation status. Two-sided Chi-Square, Student's t, and Mann-Whitney U tests were used.

**Characteristics**	**Levels**	**Total**	**BRAF wild type**	**BRAF mutation**	***p*-value**
**Number**		469	321 (68.4%)	128 (31.6%)	
**Management**					
Risk Group	Low	275 (58.6%)	240 (74.8%)	35 (23.6%)	<0.001
	Intermediate	137 (29.2%)	54 (16.8%)	83 (56.1%)	
	High	27 (8.4%)	30 (20.3%)	57 (12.2%)	
RAI treatment	Positive	167 (35.6%)	109 (34.0%)	58 (39.2%)	0.27
**Pathological data**					
Tumor type	PTC	427 (91.0%)	282 (87.9%)	145 (98%)	0.001
	FTC	25 (5.3%)	25 (7.8%)	0 (0%)	
	PTC+FTC	17 (3.6%)	14 (4.4%)	3 (2.0%)	
PTMC	Positive	175 (37.3%)	129 (40.2%)	46 (31.1%)	0.05
T stage	T1	324 (69.1%)	231 (72%)	93 (62.8%)	0.052
	T2	67 (14.3)	47 (14.6%)	20 (13.5%)	
	T3	69 (14.7)	38 (11.8%)	31 (20.9%)	
	T4	9 (1.9)	5 (1.6%)	4 (2.7%)	
N stage	N0	360 (76.8%)	274 (85.4%)	86 (58.1%)	<0.001
	N1	109 (23.2%)	47 (14.6%)	62 (41.9%)	
M stage	M0	453 (96.6%)	311 (96.9%)	142 (95.9%)	
	M1	16 (3.4%)	10 (3.1%)	6 (4.1%)	0.60
	Bilateral	127 (27.1%)	69 (21.5%)	58 (39.2%)	
Extrathyroidal extension	Positive	54 (11.5%)	21 (6.5%)	33 (22.3%)	<0.001
Angioinvasion	Positive	48 (10.2%)	35 (10.9%)	13 (8.8%)	0.48
Perineural invasion	Positive	5 (1.1%)	3 (0.9%)	2 (1.4%)	0.68
Capsular invasion	Positive	126 (26.9%)	84 (26.2%)	42 (28.4%)	0.61
Extra nodal extension	Positive	39 (8.3%)	16 (5.0%)	23 (15.5%)	<0.001
Central lymph node metastasis	Positive	106 (22.6%)	48 (15%)	58 (39.2%)	<0.001
Lateral lymph node metastasis	Positive	65 (13.9%)	30 (9.3%)	35 (23.6%)	<0.001
Hashimoto thyroiditis	Positive	140 (29.9%)	96 (29.9%)	44 (29.7%)	0.96
**Outcomes**					
Recurrence	Negative	426 (90.8%)	300 (93.5%)	126 (85.1%)	0.004
	Positive	43 (9.2%)	21 (6.5%)	22 (14.9%)	
